# Identification of anticancer OATP2B1 substrates by an in vitro triple-fluorescence-based cytotoxicity screen

**DOI:** 10.1007/s00204-019-02417-6

**Published:** 2019-03-12

**Authors:** Tímea Windt, Szilárd Tóth, Izabel Patik, Judit Sessler, Nóra Kucsma, Áron Szepesi, Barbara Zdrazil, Csilla Özvegy-Laczka, Gergely Szakács

**Affiliations:** 1 Institute of Enzymology, Research Centre for National Sciences, HAS, Budapest, 1117 Hungary; 2 Department of Pharmaceutical Chemistry, Division of Drug Design and Medicinal Chemistry, University of Vienna, Vienna, Austria; 3 Institute of Cancer Research, Medical University Vienna, Vienna, Austria

**Keywords:** High-throughput screen, Anticancer drugs, Cytotoxicity, ATP-binding cassette transporters, Organic Anon Transporting Polypeptides

## Abstract

Membrane transporters play an important role in the absorption, distribution, metabolism and excretion of drugs. The cellular accumulation of many drugs is the result of the net function of efflux and influx transporters. Efflux transporters such as P-glycoprotein/ABCB1 have been shown to confer multidrug resistance in cancer. Although expression of uptake transporters has been confirmed in cancer cells, their role in chemotherapy response has not been systematically investigated. In the present study we have adapted a fluorescence-based cytotoxic assay to characterize the influence of key drug-transporters on the toxicity of approved anticancer drugs. Co-cultures of fluorescently labeled parental and transporter-expressing cells (expressing ABCB1, ABCG2 or OATP2B1) were screened against 101 FDA-approved anticancer drugs, using a novel, automated, triple fluorescence-based cytotoxicity assay. By measuring the survival of parental and transporter-expressing cells in co-cultures, we identify those FDA-approved anticancer drugs, whose toxicity is influenced by ABCB1, ABCG2 or OATP2B1. In addition to confirming known substrates of ABCB1 and ABCG2, the fluorescence-based cytotoxicity assays identified anticancer agents whose toxicity was increased in OATP2B1 expressing cells. Interaction of these compounds with OATP2B1 was verified in dedicated transport assays using cell-impermeant fluorescent substrates. Understanding drug-transporter interactions is needed to increase the efficacy of chemotherapeutic agents. Our results highlight the potential of the fluorescence-based HT screening system for identifying transporter substrates, opening the way for the design of therapeutic approaches based on the inhibition or even the exploitation of transporters in cancer cells.

**Electronic supplementary material:**

The online version of this article (10.1007/s00204-019-02417-6) contains supplementary material, which is available to authorized users.

## Introduction

Plasma membrane proteins play important roles in regulating the cellular uptake and efflux of nutrients, xenobiotics and drugs. Expressed in pharmacological barriers, transporters modulate the absorption, distribution, metabolism and excretion (ADME) of compounds (Szakács et al. 2008; César-Razquin et al. 2015). Drug exporters such as ABCB1 (P-glycoprotein/Pgp), ABCG2 (Breast cancer resistance protein, BCRP) or ABCC1 (Multidrug resistance-associated protein 1, MRP1) belong to the ATP-binding cassette (ABC) superfamily, and typically mediate the transmembrane transport of a wide variety of xenobiotics, drugs, lipids, sterols, and metabolic products by utilizing the energy of ATP hydrolysis (Ambudkar et al. 1999; Fletcher et al. 2010). Transporters mediating drug import are members of the solute carrier (SLC) superfamily. SLC transporters also influence drug disposition and efficacy, and are sites of drug–drug interactions leading to toxicity (Giacomini et al. 2010; Giacomini and Huang 2013). One of the major SLC uptake transporters involved in ADME processes are members of the OATP (organic anion transporting polypeptide, SLCO) family. OATPs are anion exchangers mediating the cellular uptake of organic compounds with a molecular weight of more than 300 Da in an ATP- and Na^+^ independent manner (Roth et al. 2012). The liver-specific OATP1B1 (SLCO1B1) and OATP1B3 (SLCO1B3) are responsible for the hepatic detoxification of numerous drugs. According to recent FDA (US Food and Drug Administration) and EMA (European Medicines Agency) recommendations, these uptake transporters must be investigated for interaction with any novel molecular entity undergoing hepatic clearance (Giacomini and Huang 2013). OATP2B1 (SLCO2B1) is also a multispecific transporter, mediating cellular uptake of a plethora of clinically applied medicines (e.g., statins, antihistamines, and antivirals) (Tamai and Nakanishi 2013; Yu et al. 2017). However, in contrast to the members of the OATP1B subfamily, the role of OATP2B1 in hepatic clearance has not yet been proven. Owing to its wider tissue expression, OATP2B1 may participate in drug absorption from the intestine or across the blood–brain barrier (Nakanishi and Tamai 2012; Gao et al. 2015).

In the last decade, several studies revealed that the multidrug resistance phenotype in tumors is associated with the overexpression of certain ABC transporters, termed multidrug resistance (MDR) proteins. Expressed in cancer cells, ABCB1, ABCG2 or ABCC1 mediate the efflux of chemotherapeutics, allowing the survival of multidrug-resistant clones despite toxic chemotherapy (Gottesman et al. 2002). While OATP2B1 overexpression has been demonstrated in various tumors (e.g., breast cancer, gliomas, bone cysts), its role in chemotherapy response (sensitivity) has not been proven (Matsumoto et al. 2015; Kovacsics et al. 2017). In particular, the range of OATP2B1 substrates among currently used chemotherapeutics is not known. In the present study, we have adapted a fluorescence-based cytotoxic assay to identify the influence of key drug transporters on the toxicity of approved anticancer drugs. By measuring the survival of parental and transporter-expressing cells in co-cultures exposed to the drugs included in the NCI DTP oncology drug set, we identify those FDA-approved anticancer drugs, whose toxicity is influenced by the drug transporters.

## Methods

### Cell lines and culture conditions

A431 and Mes-Sa cell lines, including Mes-Sa/Dx5 were obtained from ATCC. ABCB1 and ABCG2 were expressed in A431 and Mes-Sa cells using lentiviral transduction (Pape et al. 2015), while OATP2B1 expressing cells were generated as described previously (Patik et al. 2018). After the transduction, cell lines were tested by flow cytometry and were sorted based on transporter expression/function, and in the case of the OATP2B1-expressing cell line, on CD4 protein expression. Parental, SLCO2B1, ABCG2 and ABCB1 overexpressing cell lines were transduced with the fluorescent protein expressing lentiviral supernatants produced with pRRL-EF1-mCherry, -mOrange or -eGFP expression plasmid in a second round of transduction. Mes-Sa/Dx5-eGFP cells were repeatedly cultured in 500 nM doxorubicin to ensure homogenous expression of Pgp. All cell lines were cultured at 37 °C, 5% CO_2_, in Dulbecco’s Modified Eagle’s Medium (DMEM, SigmaAldrich, 52100047), supplemented with 10% FBS, 2 mM L-glutamine and 100 units/mL penicillin and 100 µg/mL streptomycin (ThermoFisher). Functional expression of ABCB1 and ABCG2 was regularly checked with cytotoxicity assays using mitoxantrone in the absence or presence of tariquidar. Functional expression of OATP2B1 in A431-2B1 was verified by the Cascade Blue uptake assay (Patik et al. 2018) in the presence or the absence of estrone-3-sulfate (Fig. S1).

### Fluorescence-based cytotoxicity assays

All experiments were performed using a robotic pipetting workstation (Hamilton Robotics STAR Let). Cells were seeded at a final cell density of 1875 cells per well in 384-well plates in the triple co-culture (625 cells from each lines) and at 2500 cells per well in the A431 control and A431-2B1 co-culture (1250 cells from both) systems. Co-cultured cells were incubated for 24 h at 37 °C and 5% CO_2_ under humidified atmosphere prior to the addition of drugs. In the primary assays, compounds were added in a six-point dose response format with threefold dilution (the final volume was 60 µl, DMSO did not exceeded 1%). Six compounds (valrubicin, doxorubicin, daunomycin, mitoxantrone, mithramycin, dactinomycin) were excluded from the fluorescence-based screens because of their intrinsic fluorescence. 144 h after drug addition, fluorescence was recorded by a Perkin Elmer EnSpire microplate reader (GFP: 485_ex_/510_em_; mCherry: 585_ex_/610_em_; mOrange: 545_ex_/567_em_) by scanning the plate at a resolution of four points per well. Data were exported to a custom-made program for determining growth inhibition curves for each cell line. In confirmatory screens, compounds were tested with the same method in a nine-point dose response format. For validation, compounds were confirmed in cytotoxicity assays using the PrestoBlue cell viability reagent (ThermoFisher), according to the manufacturer’s instructions.

### Data analysis

In the primary screening, IC_50_ was estimated by the intercept of the linear regression fitted on nearest values. In the confirmatory screening, IC_50_ values were obtained by sigmoidal curve fitting using the GraphPad Prism software. Differences of the IC_50_ values between the cell lines were analyzed by two-sided unpaired Student *t* test, and results were considered statistically significant at a *P* value of < 0.05 (*) or 0.01 (**). Mean IC_50_ values were calculated as the average of 3–10 replicates. The Resistance Ratio (RR) was calculated by dividing the IC_50_ values measured against the multidrug resistant, transporter-expressing derivative by the cytotoxicity measured in the parental cell line; the Selectivity Ratio (SR) is the inverse of RR. Differences were considered to be biologically relevant at RR > 3 or SR > 3.

### Microplate-based uptake assay

OATP-expressing A431 cells were seeded (7 × 10^4^ cells in 200 µl final volume/well) onto 96-well plates and cultured for 16–24 h at 37 °C, 5% CO_2_. After reaching confluence, the supernatant was removed and the cells were washed three-times with 200 µl of phosphate-buffered saline (PBS). The cells were preincubated in the presence of the compounds for 5 min at 37 °C. The amount of solvent was kept below 0.5% throughout the study to avoid interference with the fluorescence of the dyes. The reaction was started with the addition of 50 µl Cascade Blue fluorescent dye (10 µM final concentration in a final volume of 100 µl) and the plate was incubated at 37 °C for 30 min (Patik et al. 2018). The reaction was stopped by the addition of 200 µl ice-cold PBS. The supernatant was rapidly removed, and the cells were washed three-times with 200 µl ice-cold PBS. Finally, 200 µl PBS was added to the cells and fluorescence was measured at room temperature using an EnSpire fluorescent plate reader (Perkin Elmer) at wavelengths 401_ex_/419_em_ nm.

### Determination of Cascade Blue dye uptake by flow cytometry

A431 cells were collected after trypsinization (0.1% trypsin) and washed twice with uptake buffer (125 mM NaCl, 4.8 mM KCl, 1.2 mM CaCl_2_, 1.2 mM KH2PO_4_, 12 mM MgSO_4_, 25 mM MES, and 5.6 mM glucose, with the pH adjusted to 5.5 using 10 N NaOH/1 M HEPES). 5 × 10^5^ cells were preincubated at 37 °C with or without estrone-3-sulfate. After preincubation, 5 µM Cascade Blue hydrazide was added in a final volume of 100 µl for 30 min. The reaction was stopped by the addition of 1 ml ice-cold PBS. The cells were kept on ice until flow cytometry analysis. The cellular fluorescence of min. 20,000 live cells was determined using an Attune Acoustic Focusing Cytometer (Applied Biosystems, Life Technologies, Carlsbad, CA, US).

### NCI DTP database and in silico screening

For in silico calculations, we focused on the NCI DTP oncology set IV compound collection and the associated cytotoxicity data released in December, 2016 (https://dtp.cancer.gov/). Putative substrates were identified based on correlation of cytotoxicity patterns to transporter expression within the NCI60 panel (Szakács et al. 2004).

## Materials

### Chemicals

The NCI DTP oncology drug set IV, containing 101 FDA-approved anticancer drugs, was obtained from the NCI/NIH DTP Open Chemical Repository as 10 mM DMSO solutions. Compounds for the follow up experiments were purchased from several vendors: methotrexate (NSC-740), teniposide (NSC-122819) and thioplex (NSC-6396) were obtained from Merck Irinotecan (NSC-616348), capecitabine (NSC-712807), bleomycin (NSC-125066), docetaxel (NSC-628503) and carfilzomib (NSC-758252) were purchased from SelleckChem; carboplatin (NSC-241240) was from Accord Healthcare; Etoposide (NSC-141540) was purchased from TEVA; estrone-3-sulfate and Cascade Blue hydrazide were from ThermoFisher Scientific.

## Results

### Establishment and validation of the triple co-cultured cell cytotoxicity assay

**Fig. 1:**
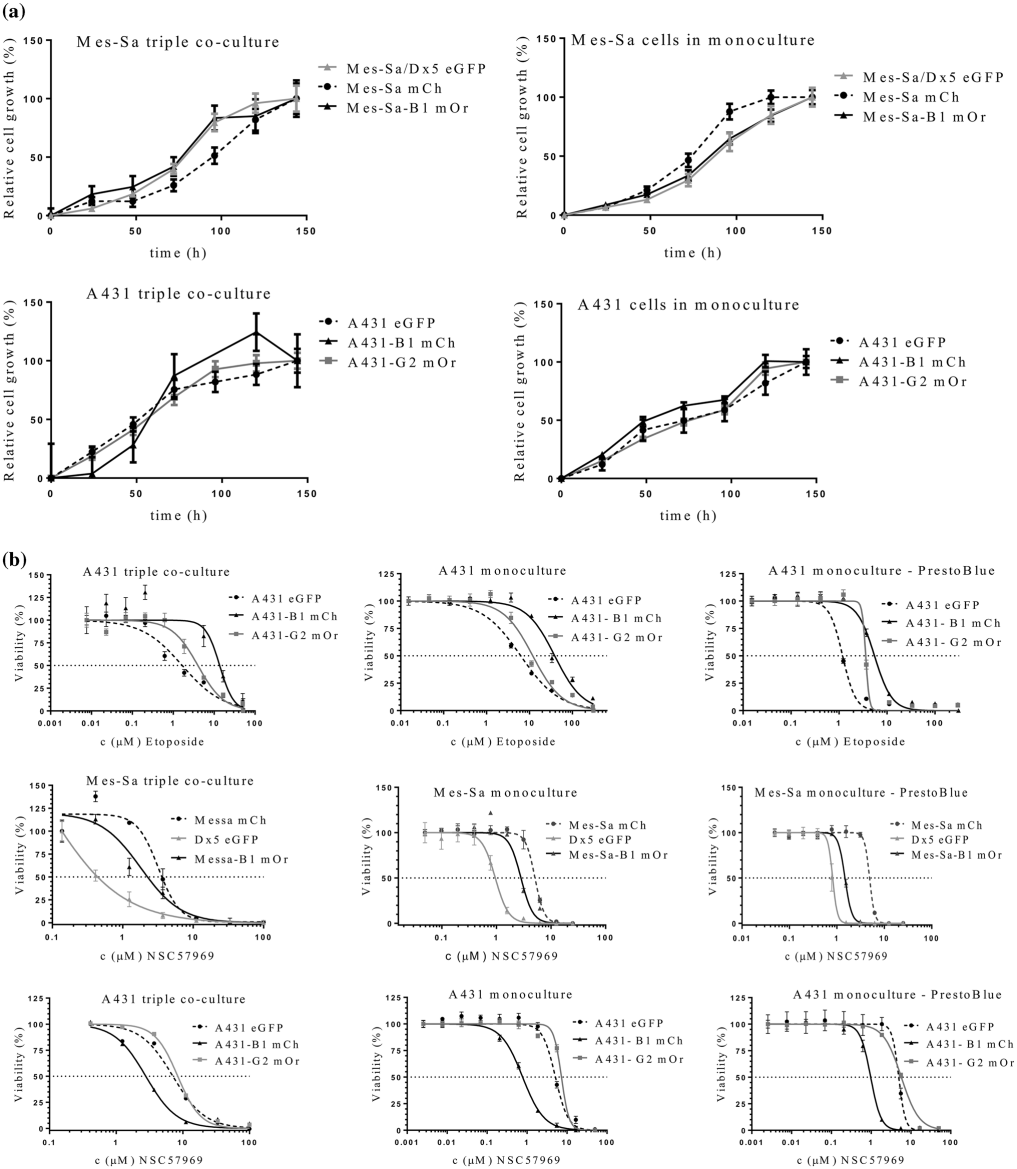
Cell growth can be reliably monitored based on the fluorescence of differently tagged cell lines. **a** Cells were plated in a 384 well plate at a final cell density of 1875 cells per well in the triple co-culture system (625 cells from Mes-Sa mCh/Mes-Sa Dx5 eGFP/ Mes-Sa-B1 mOr cell lines; 625 cells from A431 eGFP/A431-B1 mCh/A431-G2 mOr cell lines) and at 2500 cells per well in monocultures. Fluorescence corresponding to the density of the individual cell lines was recorded at regular intervals using a plate reader. **b** Validation of the triple-co-cultured fluorescence-based cytotoxicity assay. IC_50_ values measured in triple coculture condition and in monocultures. Mes-Sa, Mes-Sa-B1, Mes-Sa/Dx5 and A431, A431-B1, A431-G2 cells were seeded in 384 well plates and were incubated in the presence of increasing concentrations of etoposide or the MDR-selective compound NSC57969 in monoculture (left) and triple co-culture conditions (middle) measured with fluorescence-based cytotoxic assay, and with the PrestoBlue-based cytotoxicity assay (right). The fluorescence-based assay measured in triple coculture condition and in monoculture, compared with PrestoBlue-based cytotoxic assay provided concordant IC_50_ results (see Table S1)

We have shown earlier that the human epidermoid carcinoma cell line A431 provides a reliable and stable model for the characterization of the function of MDR ABC transporters ABCB1 and ABCG2 (Elkind et al. 2005; Nerada et al. 2016). For the purpose of this study we expressed the eGFP fluorescent protein in parental A431 cells, and mCherry or mOrange in A431 cells stably expressing ABCB1/P-glycoprotein (Pgp) or ABCG2, respectively. In addition, we transfected the human sarcoma cell line Mes-Sa and its multidrug-resistant counterparts (Mes-Sa/Dx5 and Mes-Sa-B1) with mCherry (mCh), eGFP or mOrange (mOr), respectively. Transporter activity and the stable expression of the fluorescent tags were verified by functional assays and cytotoxicity experiments (Fig. S1). Correlation between the fluorescence intensity and cell number was maintained in the triplicate co-cultures, indicating that the relative number of the parental and transporter-expressing cells can be reliably estimated by measuring the fluorescence of the individual cell lines in the same well (Fig. S2). Growth of the differently tagged cell lines could be selectively quantified both in mono- and triple co-cultures, indicating that this setup was amenable to a fluorescence-based cytotoxicity assay (Fig. 1a). The triple-fluorescence-based cytotoxicity assay measures the growth inhibitory effect of test compounds on the co-culture of differently tagged parental and transporter-expressing cell lines. The assay was validated using etoposide, which is known to be recognized by both ABCB1 and ABCG2, as well as an MDR-selective compound (NSC57969), which preferentially kills Pgp-expressing cells (Füredi et al. 2017) (Fig. 1b). The PrestoBlue-based cytotoxicity assay and the fluorescence-based assays measured in monoculture and in triple-co-culture provided concordant results, confirming that the triple-fluorescence assay is suitable for the identification of ABC transporter substrates and MDR-selective compounds (Table S1). Interestingly, despite the overlapping substrate specificity of ABCB1 and ABCG2, ABCG2 did not sensitize cells to NSC57969.

### The triple-fluorescence-based cytotoxicity assay identifies ABC transporter substrates in the DTP oncology set IV

**Fig. 2:**
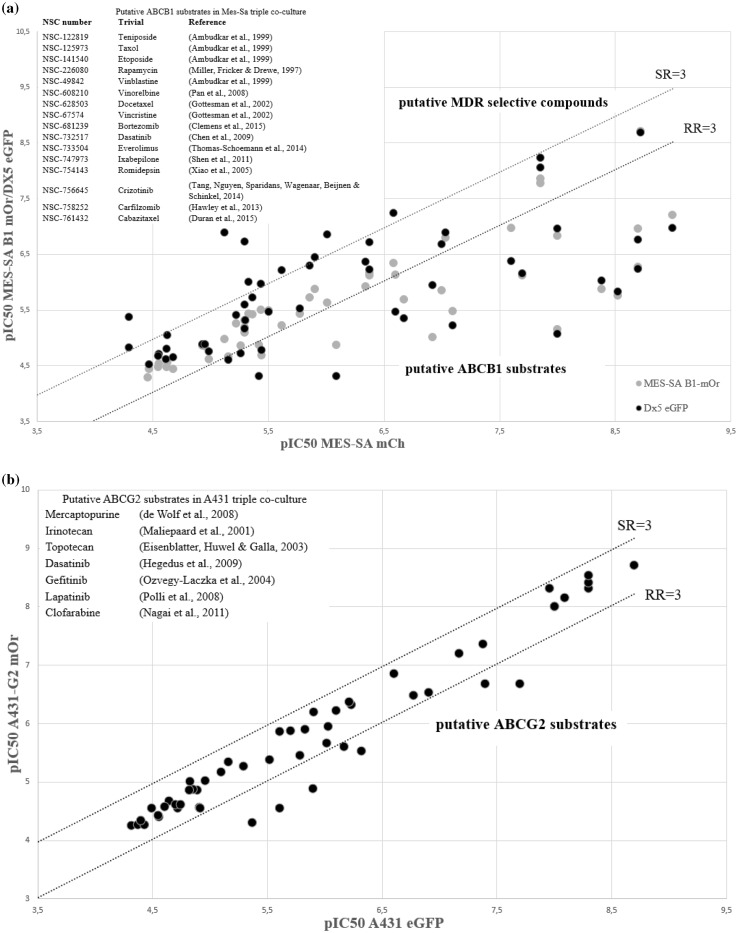
Screening results of the DTP oncology set IV measured against **a** parental Mes-Sa mCh and Pgp-expressing MDR derivatives [Mes-Sa/Dx5 eGFP (gray dots) or Mes-Sa-B1 mOr (black dots)]; **b** parental A431 and its ABCG2-expressing derivative (A431-G2). Data points represent average pIC_50_ values derived from at least two independent experiments. To evaluate the selective toxicity of the compounds, resistance ratio (RR) was calculated by dividing the IC_50_ values measured against the multidrug resistant, transporter-expressing derivative by the cytotoxicity measured in the parental cell line, while the selectivity ratio (SR) was calculated as the inverse of RR. Compounds displaying at least a threefold difference in toxicity (dashed line) between the control and transporter-expressing cells were considered as putative ABCB1 or ABCG2 substrates (RR > 3, see inset); or putative MDR-selective compounds (SR > 3, see Table S2)

Next, we used the established co-culture model system to characterize the ability of the MDR ABC proteins to modulate the cytotoxic activity of 101 clinically used chemotherapeutics compiled in the Developmental Therapeutics Program’s (DTP) oncology set IV. In the primary assays the compounds were tested in six concentrations, using a robotic pipetting workstation. As expected, the majority of the compounds were highly toxic. As compared to the parental Mes-Sa cells, approximately 20% of the oncology set compounds showed reduced toxicity against its multidrug resistant derivatives; compounds showing reduced toxicity to Mes-Sa/Dx5 cells were also less toxic to Mes-Sa/B1 cells, proving that these compounds are P-glycoprotein substrates (Fig. 2a, Table S3). Interestingly, several compounds including NSC-125066 (Bleomycin), NSC-9706 (Triethylenemelamine), NSC-6396 (Thioplex), and NSC-762 (Mechlorethamine) seemed to be more toxic against Mes-Sa/Dx5 cells. However, none of these compounds proved to preferentially kill Mes-Sa-B1 cells, indicating that the paradoxical sensitivity of Mes-Sa/Dx5 cells is independent of Pgp (Table S2). To substantiate these results, we repeated the screen using the co-culture of A431 cells. Putative Pgp substrates identified by the screen performed by the Mes-Sa cells showed reduced toxicity to A431-B1 mCh cells (Table S3), but the collateral sensitivity of Mes-Sa/Dx5 cells could not be reproduced (Fig. S3). Next, we analyzed the differential sensitivity of parental and ABCG2-expressing A431 cells. Similarly to Pgp, ABCG2 conferred resistance to several compounds, which included known ABCG2-substrates: mercaptopurine (Wolf et al. 2008), irinotecan, topotecan (Maliepaard et al. 2001), dasatinib, gefitinib, lapatinib (Ozvegy-Laczka et al. 2004; Hegedus et al. 2009), clofarabine (Nagai et al. 2011) (Fig. 2b). On the other hand, none of the compounds were found to be more toxic to ABCG2-expressing cells.

### The fluorescence-based cytotoxicity assay identifies putative OATP2B1 transporter substrates

**Fig. 3:**
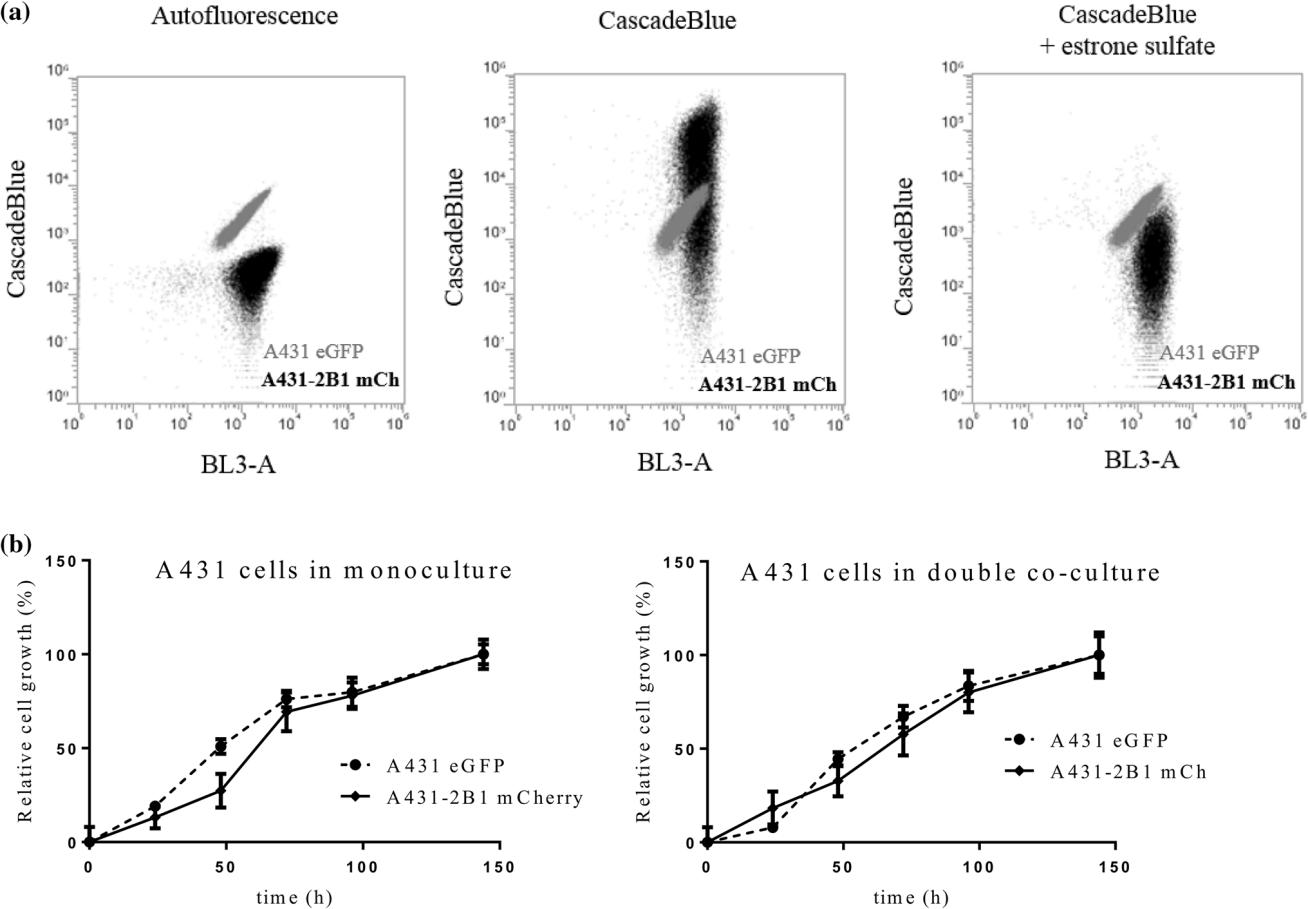
Characterization of the fluorescence-based OATP2B1 uptake transporter-expressing A431 co-culture system. **a** Transport activity of OATP2B1 can be selectively measured in the co-cultured cells. Co-culture of parental A431 eGFP (grey) and transporter expressing A431-2B1 mCh (black) cells were stained by Cascade Blue in the absence (middle) or presence (right) of the OATP2B1 inhibitor estrone-3-sulfate (cells without Cascade Blue are shown in the left panel). CB is selectively taken up by OATP2B1-expressing cells. **b** Growth of A431 eGFP and A431-2B1 mCh cells can be reliably monitored based on the differential tagging of cells. Cells were seeded in 384-well plates in monoculture (2500 cells/well) and in co-culture conditions (1250 A431 eGFP + 1250 A431-2B1 cells/well). The cells were incubated for 144 h in a final volume of 60 µl, at 37 °C and 5% CO_2_, under humidified atmosphere. Cell growth corresponding to each cell line was recorded using a microplate reader

Our experiments performed with the co-culture systems indicated that the fluorescent protein based screen can identify ABCB1 and ABCG2 substrates. Recent evidence suggests that uptake transporters can play a significant role in the uptake of cytotoxic drugs. However, compared to MDR transporters, there are no systematic studies addressing the contribution of uptake transporters to cytotoxicity. Recently, we have established a panel of cell lines expressing the members of the OATP transporter family, OATP1B1, 1B3 and 2B1, and shown that A431 cells can be used to study the function of these OATP transporters (Patik et al. 2018). To address the ability of OATP2B1 to modulate the toxicity of the DTP compound set, we labeled OATP2B1 expressing A431 cells with mCherry. Transporter activity and the fluorescence of the cell lines were verified by FACS analysis (Figs. 3a, S1). Growth curves of the co-cultures confirmed that the relative number of the parental and OATP2B1-expressing cells can be reliably estimated (Fig. 3b), suggesting that the fluorescence-based co-culture system can be used to characterize the sensitivity of OATP2B1 expressing cells.

**Fig. 4:**
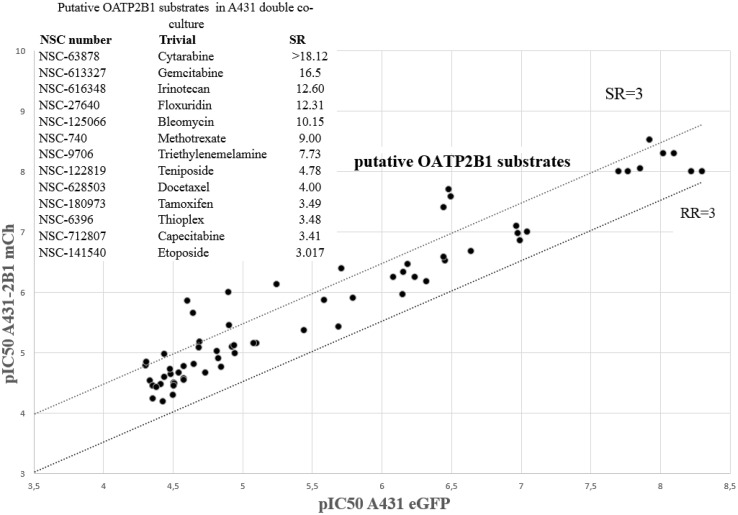
Screening results of the DTP Oncology Set IV measured against parental A431 and its OATP2B1-expressing derivative (A431-2B1). Compounds displaying at least a threefold difference in toxicity (dashed line) between the control and transporter-expressing cells were considered as putative substrates (SR > 3, see inset)

In the case of OATP2B1, which mediates the uptake of large, organic molecules, including chemotherapeutic compounds (Thakkar et al. 2015), we expected an increased toxicity of transporter substrate chemotherapeutics, due to their elevated accumulation in the cells. Whereas the majority of the compounds were equally toxic to A431eGFP and A431-2B1 cells, 13 approved chemotherapeutics showed increased activity against A431 cells expressing the uptake transporter (Fig. 4). Two compounds that showed uncharacteristic dose–response patterns were not pursued further (NSC-63878, NSC-613327), one compound (NSC-9706) was unavailable, and another compound was removed due to intrinsic fluorescence (NSC-757441). Increased toxicity of the remaining compounds against OATP2B1 expressing cells was confirmed in additional cytotoxicity experiments (Table S4), indicating that these compounds may be transported by OATP2B1 into the cells. To test this proposition, we evaluated the effect of these compounds on the accumulation of CascadeBlue in A431-2B1 cells. The results clearly indicated that the putative OATP2B1-substrates identified by the fluorescence-based cytotoxicity screen show high affinity interaction with OATP2B1 (Table 1).

**Table 1:** Inhibition of OATP2B1-mediated Cascade Blue (CB) uptake by the putative OATP2B1 substrates identified in the cytotoxicity screen

NSC number	Name	IC_50_ (µM)
NSC-125066	Bleomycin	64.15 ± 6.1
NSC-712807	Capecitabine	124.01 ± 6.42
NSC-628503	Docetaxel	11.32 ± 0.38
NSC-616348	Irinotecan	17.03 ± 1.46
NSC-180973	Tamoxifen	10.99 ± 0.7
NSC-740	Methotrexate	265.50 ± 31.38
NSC-122819	Teniposide	4.59 ± 0.4
NSC-141540	Etoposide	1.88 ± 0.32
NSC-6396	Thioplex	574.28 ± 282.7

Transport of CB (10 µM) was measured for 30 min in increasing concentrations of the investigated compounds. IC_50_ values were determined by measuring the intracellular accumulation of Cascade Blue in the presence of increasing concentrations of OATP2B1 interacting compounds. Experiments were performed in triplicates with three parallels in each biological replicate. Average ± SD values of IC_50_ values are shown

## Discussion

Drug transporters are widely acknowledged as important determinants of drug pharmacokinetics, pharmacodynamics, and toxicity, underlying drug–drug interactions, lack of therapeutic efficacy or unwanted toxicity. A large variety of in vitro assays have been used to characterize the interaction of compounds with transporters (Polli et al. 2001; Sarkadi et al. 2006). While each approach has unique strengths, there are also limitations, and classification of compounds as transporter substrates or inhibitors often depends on the specific assay.

Whereas pharmacological models based on, e.g., MDCK or CaCo-2 cells address the role of transporters in pharmacological barriers such as the gut or the blood–brain barrier, here we wanted to evaluate the influence of the transporters on the chemosensitivity of cancer. In this study, we characterize Mes-Sa (human sarcoma) and A431 (human epidermoid carcinoma) cells, along with their transporter-expressing derivatives. In prior work our group has shown that both cell lines are suitable for the identification of cytotoxic MDR-substrates and MDR-selective compounds (Elkind et al. 2005; Hegedűs et al. 2009; Türk et al. 2009; Nerada et al. 2016; Füredi et al. 2017; Szabó et al. 2018; Temesszentandrási-Ambrus et al. 2018). To evaluate the influence of OATP2B1 in the context of the MDR exporters, we wanted to use the same in vitro model. We found that A431 cells are particularly well suited for the evaluation of OATP function. First, A431 cells show optimal growth and adherence characteristics favoring HTS applications. Second, A431 cells allow the stable expression of OATP transporters at relevant levels. Third, the signal to noise ratio measured in the A431 cell system was particularly high, given the very low permeability of these cells to the fluorescent dyes used to probe OATP function (Patik et al. 2018).

In oncological developments, in vitro cytotoxicity testing provides a convenient means to characterize the toxicity of new chemical entities. Since cellular accumulation of most drugs is significantly influenced by the net function of efflux and influx transporters, cytotoxicity of the compounds also depends on their interaction with transporters. Thus, cytotoxicity assays performed with pairs of parental and transporter-expressing cells can be used to identify transporter substrates. In the case of efflux pumps, compounds that are recognized for transport are expected to show reduced toxicity. In contrast, uptake transporters may increase the toxicity of their cytotoxic substrates by enhancing their cellular accumulation. The toxicity of many chemotherapeutics, including new-generation anticancer agents, are known to be blunted by efflux transporters, but the influence of uptake transporters is relatively unexplored. To improve the throughput of transporter-based cytotoxicity screens, Brimacombe et al. introduced a dual-fluorescence system in which they compared the sensitivity of the OVCAR-8 cell line with that of its drug-selected derivative NCI/ADR-RES (expressing ABCB1/Pgp), allowing sensitive and resistant cells to be treated with compounds under shared conditions in the same wells (Brimacombe et al. 2009). Here we take this approach a step further to include up to three cell lines expressing two major efflux pumps and a ubiquitous uptake transporter.

The fluorescence-based viability assay is based on the co-culture of differentially tagged cell lines. To achieve stable expression of transporters, ABCB1 and ABCG2 were expressed in A431 and Mes-Sa cells by lentiviral transduction, while SLCO2B1 expressing cells were established by the Sleeping Beauty transposon-based gene delivery system, using the 100*×* hyperactive SB transposase. In a second round of transduction, the cell lines were transduced with the fluorescent protein expressing lentiviral supernatants encoding mCherry, mOrange or eGFP, carefully chosen to allow parallel detection of the cell lines in the same well. Using a robotic work-flow, we were able to increase the throughput of our assay to include DTP’s approved oncology set, containing most current FDA-approved anticancer drugs (Table S5). We developed three parallel cellular models to evaluate the ability of the triple-fluorescence assay to capture known ABCB1/Pgp substrates. We compared the sensitivity of the parental Mes-Sa cells with that of its drug-selected derivative (Mes-Sa/Dx5), and Mes-Sa-B1 cells, which were engineered to overexpress Pgp. In addition, we characterized the sensitivity of parental A431 cells in comparison to A431 cells expressing ABCB1 or ABCG2. Results obtained with ABCB1-overexpressing cells confirmed that the triple co-culture cytotoxicity assay identifies P-glycoprotein substrates. Overall, the three P-gp positive cell lines gave similar results—however, resistance mediated by ABCB1 was not identical in the three cell lines (Table S3). The Oncology Set has also been measured across the NCI60 cell panel, and it has been shown that the toxicity pattern of ABCB1/Pgp substrates is negatively correlated with the pattern of ABCB1 expression across the NCI60 cell panel (Alvarez et al. 1995; Szakács et al. 2004). As shown in Fig. S4a, all compounds identified as putative Pgp substrates by the Mes-Sa triple co-culture assay are characterized by a negative Pearson correlation coefficient (PCC < − 0.4), indicating that the fluorescence-based triple co-culture cytotoxicity assay can be cross-validated by data from the NCI-60 screen (similar results were obtained by the A431 panel) (Fig. S4c). At the same time the in silico prediction missed several known Pgp substrates including vincristine, dasatinib, docetaxel, ixabepilone, cabazitaxel, teniposide and etoposide, which were identified by both the Mes-Sa and A431 co-culture assays (Table S4). Dasatinib, which was reported to be a substrate of both P-gp and ABCG2, was less toxic to all MDR cells. Conversely, crizotinib and bortezomib, both described as Pgp substrates, was only identified by the Mes-Sa panel (RR = 9.9 and 4.3 for Mes-Sa-B1 and RR = 21.3 and 17 for Mes-Sa/Dx5, respectively), while the resistance ratio of these compounds were below the established threshold in the A431 cells (RR = 2.6 and 2.7, respectively). Interestingly, the DTP database contains toxicity patterns that are positively correlated to the expression of ABCB1 across the NCI60 cell panel, indicating that Pgp may potentiate, rather than reduce the toxicity of certain compounds (Szakács et al. 2004). Indeed, compounds such as NSC57969 show preferential toxicity to Pgp-expressing cells, proving that this well-studied drug resistance mechanism may be exploited to target resistant cancer (Hall et al. 2009). However, as seen in Figs. 2a and S3, the paradoxical toxicity of the compounds is restricted to Mes-Sa/Dx5 cells, indicating that their increased toxicity is unrelated to Pgp (Szakács et al. 2014).

Increasing evidence indicates that OATPs show altered, usually increased expression in various types of cancer, e.g., in breast, colon, lung and prostate cancers (Obaidat et al. 2012; Thakkar et al. 2015; Kovacsics et al. 2017). Increased OATP2B1 expression has been demonstrated in a colon cancer cell line (Tamai et al. 2000), in bone cysts (Liedauer et al. 2009) and in glioma (Bronger et al. 2005). OATP overexpression may be beneficial for tumor cells by increased nutrient or hormone uptake. Indeed, it has been shown that increased uptake of hormones (ES, DHEAS) by OATPs 1A2, 1B3 and 2B1 resulted in increased survival of breast cancer cells (Nozawa et al. 2004; Arakawa et al. 2012; Matsumoto et al. 2015). Moreover, clinical data suggests that testosterone uptake by OATP1B3 or DHEAS transport by OATP2B1 may be crucial in prostate cancer progression (Hamada et al. 2008; Wright et al. 2011). Therefore, inhibition of OATP function presents a possible strategy to cure hormone dependent cancers.

On the other hand, multispecific OATPs, such as OATP1A2, 1Bs and 2B1 may also influence the in vivo fate of drugs as well as their intratumoral concentration. Hence, increased cellular uptake of anticancer compounds by these OATPs may lead to increased drug sensitivity, offering a strategy to increase the efficacy of chemotherapy. To date, only few studies have investigated the role of OATPs in drug sensitivity. OATP1B1 has been shown to sensitize cells toward flavopiridol (Brenner et al. 2015), and OATP1B3 and OATP2B1 were shown to increase the toxicity of methotrexate and atorvastatin, respectively (Abe et al. 2001). On the analogy of efflux transporters, correlations were found between drug activity and the mRNA expression of SLC transporters across the NCI60 cell panel (Okabe et al. 2008; Lancaster et al. 2013). However, besides the above mentioned in silico screen, a systemic search for OATP-mediated cytotoxicity has not been undertaken. After validating the fluorescence-based co-culture system with traditional cytotoxicity experiments performed in monocultures (Fig. 1, Table S1), and the identification of known ABCB1 and ABCG2 substrates (Fig. 2, Table S3) we repeated the screen subjecting the co-cultures of parental and OATP2B1-expressing cells to the drug panel. In agreement with the directionality of the transport, the assay has failed to identify compounds showing reduced toxicity in transporter-expressing cells. Conversely, 13 chemotherapeutics proved to be more toxic against OATP2B1 overexpressing A431 cells, as compared to the parental line. Except for methotrexate and etoposide, none of these compounds have been reported to interact with OATP2B1. Etoposide was previously reported as substrate of OATP2B1 (Fahrmayr et al. 2012), and its close structural analog teniposide was reported to be an inhibitor for both hepatic transporters OATP1B1 and OATP1B3 (De Bruyn et al. 2013). The uniform platform introduced here allows the comparative analysis of transporter-effect on cell survival. It is interesting to note that both etoposide and teniposide are also recognized by ABCG2 (Allen et al. 2003), and etoposide is additionally recognized by ABCB1, which may mask the OATP2B1-mediated effect in tumor cells expressing several transporters. Docetaxel is also transported by all the three studied transporters, and further studies suggest interaction with OATP1B3 (Baker et al. 2009). However, it was reported that taxol is not transported by OATP2B1 (Leblanc et al. 2018), which was confirmed by our screening results (Fig. 4). We find that tamoxifen, which was reported to be a substrate of OATP1B1 (Gao et al. 2017), shows high affinity interaction with OATP2B1 (Table S5). This drug is of special interest since it is commonly used for the treatment of ER-positive breast cancer and the facilitated uptake of tamoxifen into cancer cells by uptake transporters can, therefore, possess an advantage. However, since also efflux transporters (ABCB1 and ABCG2) recognize tamoxifen, the net-effect of this uptake/efflux interplay is unknown, when both types of transporters are expressed. The clinical relevance of transporter-mediated drug uptake depends on many pathophysiological factors including expression levels or the context of efflux transporters. The results presented in this study prove that in vitro, OATP2B1 can sensitize cells to its transported substrates. Further research should evaluate the role of the interplay of transporters in drug resistant cancer, and it will be essential to fully understand the full plethora of transporters involved in drug disposition. The methodology presented here provides an experimental tool for the comparison of efflux and uptake transport mechanisms in different variants of the same cell line.

## Electronic supplementary material

Below is the link to the electronic supplementary material.

Supplementary material 1 (DOCX 407 KB)
