# Supplementary material for: Identification of anticancer OATP2B1 substrates by an in vitro triple-fluorescence-based cytotoxicity screen
Source: Arch Toxicol. 2019 Mar 12;93(4):953–64. doi: 10.1007/s00204-019-02417-6 (PMC6510822; doi:10.1007/s00204-019-02417-6)

**Identification of anticancer OATP2B1 substrates by an *in vitro* triple-fluorescence based cytotoxicity screen**

Tímea Windt^1^, Szilárd Tóth^1^, Izabel Patik^1^, Judit Sessler^1^, Nóra Kucsma^1^, Áron Szepesi^1^, Barbara Zdrazil^2^, Csilla Özvegy-Laczka^1^, Gergely Szakács^1,3,*^

^1^Institute of Enzymology, Research Centre for National Sciences, HAS, Budapest, 1117, Hungary

^2^Department of Pharmaceutical Chemistry, Division of Drug Design and Medicinal Chemistry, University of Vienna, Austria

^3^Institute of Cancer Research, Medical University Vienna, Vienna, Austria

^*^ Correspondence to Gergely Szakacs, szakacs.gergely@mta.ttk.hu, +43 (0)1 40160 - 57619

**Keywords:** High-throughput screen, anticancer drugs, cytotoxicity, efflux, uptake, transporters

**Supporting Information**

Supplementary Tables

**Table S1**. Cytotoxicity (IC_50_ values in uM) of etoposide, the MDR-selective compound NSC57969 measured by the PrestoBlue viability assay and the fluorescent protein based cytotoxicity assay in monocultures or in the triplicate A431 co-cultures (A431, A431B1 and A431G2) and the triplicate Mes-Sa co-culture (Mes-Sa, Mes-Sa/Dx5 and Mes-Sa-B1).

**Table S2:** List of compounds preferentially killing drug resistant Mes-Sa/Dx5 cells as compared to parental Mes-Sa cells (SR>3).

***Table S3****. List of compounds susceptible to Pgp-mediated resistance in Mes-Sa/Dx5, Mes-Sa/B1 and A431B1 cells. The Resistance Ratio (RR) was calculated by dividing the IC_50_ values measured against the multidrug resistant, transporter expressing derivative by the cytotoxicity measured in the respective parental cell line. The screen captured established Pgp-substrates, as indicated by the references.*

**Table S4**: Cytotoxicity (IC_50_) of compounds (µM) showing increased toxicity (SR>3) against OATP2B1-expressing A431 cells measured in co-culture (**a**) and in monoculture (**b**). Average IC50 values were calculated based on 9 concentration points, using at least 3 replicates. Differences between the IC_50_ values were analyzed by two-sided unpaired Student t test, and results were considered statistically significant at a P value of <0.05 (*) or 0.01 (**).

**Table S5**: List of compounds and concentrations used in the study.

Supplementary Figures

**Figure S1. Characterization of the cell lines**

**a)** Fluorescence intensity of the tagged A431 and Mes-Sa cell lines. The homogeneity and stability of the fluorescent cell lines were monitored using an Attune® Acoustic Focusing Cytometer (Applied Biosystems, Life Technologies, Carlsbad, CA, US) at an excitation wavelength of 488 nm. eGFP-expressing cells were detected using the BL1 channel (emission filter 530/30 nm), mCh cells were detected in the BL3 channel (emission filter 695/40 nm), whereas mOr cells were detected in BL2 (emission filter 574/26 nm).

**b-c)** Expression of ABCB1 or ABCG2 confers resistance to irinotecan (**B**) or etoposide (**C**). Resistance of A431B1 and G2 expressing cells was abolished in the presence of tariquidar.

**d**) A4312B1 cells show an Estrone-3-sulphate-dependent cascade blue (CB) uptake (*see Methods*). Uptake of CB was measured following incubation of the cells with 5 µM CB for 30 minutes (dark grey). In control experiments, cells were preincubated for 5 minutes with Estrone-3-sulphate (light grey). Autofluorescence of cells is shown in black.

**e)** Flow cytometry analysis of DyeCycle Violet (DCV) accumulation in A431 and Mes-Sa cell lines. Data analysis was performed using Attune^TM^ Nxt v2.6 Software (Invitrogen^TM^, Thermo Fischer Scientific). DCV transport experiments were carried out in uptake buffer (125 mM NaCl, 4.8 mM KCl, 1.2 mM CaCl_2_, 1.2 mM KH_2_PO_4_, 12 mM MgSO_4_, 25 mM MES, and 5.6 mM glucose, with the pH adjusted to 7.4, using 1 M HEPES). ABCB1 and ABCG2 function was investigated using 1 µM DCV (a substrate of both transporters), following incubation for 30 minutes in the absence or presence of 1 µM tariquidar (inhibitor of both transporters), which was added 5 minutes prior to DCV addition. MDR activity factor % (MAF%) was calculated as MAF% = (((MFI_inh_-MFI_0_)/MFI_inh_)×100), where MFI_inh_ and MFI_0_ are the median fluorescent intensity (MFI) with (inh) or without (0) inhibitor (Homolya, Hollo, Muller, Mechetner & Sarkadi, 1996).

**Figure S2:** Cell numbers can be reliably estimated based on the fluorescence of cells cultured in monoculture or triple co-culture conditions. Increasing number of cells were seeded either in monocultures or triple co-cultures. Fluorescence was recorded using Perkin Elmer EnSpire microplate reader. The number of eGFP (**a**), mCh (**b**) or mOr (**c**) expressing cells can be reliably estimated in triple co-cultures.

**Figure S3**: Screening results of the DTP Oncology Set measured against parental A431 and Pgp-expressing A431B1 cells using the triple fluorescence based assay. Data points represent average pIC_50_ values of at least two independent experiments. Compounds showing attenuated toxicity against A431B1 cells are listed (RR>3).

**Figure S4**: Relationship between drug sensitivity and ABCB1 expression in the NCI60 predicts ABCB1 substrates identified by the fluorescence-based screen. Pearson correlation coefficients (PCC) between the expression of ABCB1 and the activity pattern of the 101 drugs compiled in the NCI DTP Oncology Drug Set IV were calculated as described in (Szakács et al., 2004); PCC<-0,4 indicates putative substrates). Black dots represent compounds whose toxicity was uniformly attenuated by ABCB1 in the Mes-Sa triple-co-culture system (RR>3) measured against Mes-Sa-B1 **(a)**, Mes-Sa/Dx5 cell line (**b**) and A431B1 expressing cells (**c)**.

**Table S1**

|  |  |  | | |
| --- | --- | --- | --- | --- |
|  |  | triple co-culture | monoculture | PrestoBlue |
| Etoposide | A431:  A431B1:  A431G2: | 1.691  13.77  4.049 | 6.098  46.27  11.97 | 1.188  5.489  3.425 |
| NSC57969 | Mes-Sa:  Mes-Sa/DX5:  Mes-Sa-B1: | 3.208  0.177  1.799 | 5.076  0.951  2.809 | 4.891  0.815  1.482 |
|  | A431:  A431B1:  A431G2: | 7.094  2.783  8.392 | 5.127  0.774  7.257 | 4.932  0.974  5.760 |

**Table S2**

|  |  |  |  |  |
| --- | --- | --- | --- | --- |
|  | **NSC** | **Name** | **SR  (Mes-Sa/DX5)** | **SR**  **(Mes-Sa-B1)** |
|  | NSC-125066 | Bleomycin | 57.8 | 1.44 |
|  | NSC-9706 | Triethylenemelamine | 26.62 | 1.3 |
|  | NSC-6396 | Thioplex | 12.56 | - |
|  | NSC-762 | Mechlorethamine | > 11.58 | - |
|  | NSC-752 | Thioguanine | 6.93 | 0.41 |
|  | NSC-747599 | Nilotinib | 4.68 | 1.23 |
|  | NSC-26980 | Mitomycin | 4.407 | 0.56 |
|  | NSC-755 | Mercaptopurine | 3.87 | 0.39 |
|  | NSC-606869 | Clofarabine | 3.44 | 0.91 |
|  | NSC-755986 | Vismodegib | > 3.31 | - |
|  | NSC-698037 | Pemetrexed | 3.29 | 1.14 |

**Table S3**

| **NSC number** | **Trivial** | **RR  (Mes-Sa-B1)** | **RR  (A431B1)** | **RR (Dx5)** | **Reference** |
| --- | --- | --- | --- | --- | --- |
| NSC-122819 | Teniposide | 41.88 | 5.43 | 75.25 | (Ambudkar et al., 1999) |
| NSC-125973 | Taxol | 593.3 | 50.91 | 497.67 | (Ambudkar et al., 1999) |
| NSC-141540 | Etoposide | 16.97 | > 4.14 | > 61.73 | (Ambudkar et al., 1999) |
| NSC-49842 | Vinblastine | 15.00 | 244.4 | 11.00 | (Ambudkar et al., 1999) |
| NSC-608210 | Vinorelbine | 725.00 | 232.00 | 861.00 | (Pan et al., 2008) |
| NSC-628503 | Docetaxel | 275.00 | 26.38 | 295.00 | (Gottesman et al., 2002) |
| NSC-67574 | Vincristine | 38.00 | 56.8 | 36.00 | (Gottesman et al., 2002) |
| NSC-732517 | Dasatinib | 3.147 | 11.5 | > 3.60 | (Chen et al., 2009) |
| NSC-747973 | Ixabepilone | >208.3 | 23.32 | 9.66 | (Shen et al., 2011) |
| NSC-754143 | Romidepsin | 331.70 | > 97 | > 234.15 | (Xiao et al., 2005) |
| NSC-758252 | Carfilzomib | 55.50 | 47.4 | 88.00 | (Hawley et al., 2013) |
| NSC-761432 | Cabazitaxel | 63.00 | 6.00 | > 107.00 | (Duran et al., 2015) |

**Table S4**

**a**

| NSC number | Trivial name | IC_50_ (µM)  A431 eGFP | | | IC_50_ (µM) A4312B1 mCh | | | **SR** |
| --- | --- | --- | --- | --- | --- | --- | --- | --- |
| NSC-122819 | Teniposide | 0.45 | ± | 0.14 | 0.07 | ± | 0.033 | 6.02^*^ |
| NSC-125066 | Bleomycin | 7.47 | ± | 1.69 | 0.83 | ± | 0.16 | 9.01^**^ |
| NSC-141540 | Etoposide | 7.45 | ± | 2.28 | 0.82 | ± | 0.048 | 9.06^**^ |
| NSC-180973 | Tamoxifen | 9.76 | ± | 0.22 | 8.28 | ± | 0.38 | 1.18^*^ |
| NSC-616348 | Irinotecan | 13.83 | ± | 3.18 | 1.45 | ± | 0.55 | 9.51^**^ |
| NSC-628503 | Docetaxel | 0.08 | ± | 0.016 | 0.002 | ± | 0.013 | 4.98 |
| NSC-6396 | Thioplex | 57.65 | ± | 10.45 | 18.72 | ± | 4.66 | 3.08^*^ |
| NSC-712807 | Capecitabine | 205.40 | ± | 12.56 | 44.49 | ± | 3.29 | 4.62^**^ |
| NSC-740 | Methotrexate | 0.38 | ± | 0.11 | 0.03 | ± | 0.009 | 11.78^**^ |

**b**

| NSC number | Trivial name | IC_50_ (µM)  A431 eGFP | | | IC_50_ (µM) A4312B1 mCh | | | **SR** |
| --- | --- | --- | --- | --- | --- | --- | --- | --- |
| NSC-122819 | Teniposide | 0.83 | ± | 0.15 | 0.14 | ± | 0.05 | 6.1^**^ |
| NSC-125066 | Bleomycin | 16.3 | ± | 4.65 | 1.5 | ± | 0.24 | 10.9^**^ |
| NSC-141540 | Etoposide | 6.42 | ± | 1.67 | 1.99 | ± | 0.81 | 3.2^*^ |
| NSC-180973 | Tamoxifen | 11.35 | ± | 0.50 | 12.21 | ± | 0.36 | 0.9 |
| NSC-616348 | Irinotecan | 11.11 | ± | 2.61 | 2.03 | ± | 0.34 | 5.47^**^ |
| NSC-628503 | Docetaxel | 0.00097 | ± | 0.00002 | 0.00022 | ± | 0.000145 | 4.35^*^ |
| NSC-6396 | Thioplex | 57.65 | ± | 10.45 | 18.72 | ± | 4.66 | 3.08^*^ |
| NSC-712807 | Capecitabine | 390.55 | ± | 50.47 | 128.58 | ± | 18.88 | 3.04^**^ |
| NSC-740 | Methotrexate | 0.43 | ± | 0.09 | 0.12 | ± | 0.034 | 3.59^**^ |

**Table S5**

| NSC-102816 | 50 µM | to | 0,103 µM |
| --- | --- | --- | --- |
| NSC-105014 | 25 µM | to | 0,041 µM |
| NSC-109724 | 50 µM | to | 0,206 µM |
| NSC-119875 | 25 µM | to | 0,103 µM |
| NSC-122758 | 50 µM | to | 0,206 µM |
| NSC-122819 | 25 µM | to | 0,103 µM |
| NSC-125066 | 50 µM | to | 0,103 µM |
| NSC-125973 | 25 µM | to | 0,0021 µM |
| NSC-127716 | 50 µM | to | 0,206 µM |
| NSC-13875 | 50 µM | to | 0,206 µM |
| NSC-138783 | 50 µM | to | 0,206 µM |
| NSC-1390 | 50 µM | to | 0,206 µM |
| NSC-141540 | 50 µM | to | 0,103 µM |
| NSC-14229 | 25 µM | to | 0,041 µM |
| NSC-169780 | 50 µM | to | 0,206 µM |
| NSC-180973 | 25 µM | to | 0,103 µM |
| NSC-18509 | 25 µM | to | 0,103 µM |
| NSC-19893 | 50 µM | to | 0,103 µM |
| NSC-218321 | 50 µM | to | 0,206 µM |
| NSC-226080 | 50 µM | to | 0,103 µM |
| NSC-241240 | 50 µM | to | 0,206 µM |
| NSC-25154 | 50 µM | to | 0,206 µM |
| NSC-26271 | 50 µM | to | 0,206 µM |
| NSC-266046 | 50 µM | to | 0,103 µM |
| NSC-26980 | 25 µM | to | 0,0206 µM |
| NSC-27640 | 25 µM | to | 0,0021 µM |
| NSC-296961 | 50 µM | to | 0,206 µM |
| NSC-3088 | 25 µM | to | 0,103 µM |
| NSC-312887 | 50 µM | to | 0,206 µM |
| NSC-32065 | 50 µM | to | 0,206 µM |
| NSC-34462 | 50 µM | to | 0,103 µM |
| NSC-362856 | 50 µM | to | 0,206 µM |
| NSC-369100 | 50 µM | to | 0,206 µM |
| NSC-38721 | 50 µM | to | 0,103 µM |
| NSC-409962 | 50 µM | to | 0,206 µM |
| NSC-45388 | 50 µM | to | 0,206 µM |
| NSC-45923 | 50 µM | to | 0,206 µM |
| NSC-49842 | 25 µM | to | 0,0021 µM |
| NSC-606869 | 50 µM | to | 0,103 µM |
| NSC-608210 | 25 µM | to | 0,00021 µM |
| NSC-609699 | 25 µM | to | 0,206 µM |
| NSC-613327 | 25 µM | to | 0,0021 µM |
| NSC-616348 | 50 µM | to | 0,103 µM |
| NSC-628503 | 25 µM | to | 0,000041 µM |
| NSC-63878 | 25 µM | to | 0,103 µM |
| NSC-6396 | 50 µM | to | 0,206 µM |
| NSC-66847 | 25 µM | to | 0,103 µM |
| NSC-67574 | 25 µM | to | 0,0021 µM |
| NSC-681239 | 25 µM | to | 0,0021 µM |
| NSC-686673 | 50 µM | to | 0,206 µM |
| NSC-698037 | 50 µM | to | 0,103 µM |
| NSC-701852 | 25 µM | to | 0,103 µM |
| NSC-702294 | 50 µM | to | 0,206 µM |
| NSC-712807 | 50 µM | to | 0,206 µM |
| NSC-713563 | 50 µM | to | 0,103 µM |
| NSC-71423 | 50 µM | to | 0,206 µM |
| NSC-715055 | 25 µM | to | 0,0021 µM |
| NSC-718781 | 25 µM | to | 0,041 µM |
| NSC-719276 | 50 µM | to | 0,103 µM |
| NSC-719344 | 50 µM | to | 0,206 µM |
| NSC-719345 | 50 µM | to | 0,206 µM |
| NSC-719627 | 50 µM | to | 0,103 µM |
| NSC-721517 | 50 µM | to | 0,206 µM |
| NSC-732517 | 25 µM | to | 0,0041 µM |
| NSC-733504 | 25 µM | to | 0,103 µM |
| NSC-737754 | 25 µM | to | 0,103 µM |
| NSC-740 | 25 µM | to | 0,0021 µM |
| NSC-743414 | 50 µM | to | 0,206 µM |
| NSC-745750 | 25 µM | to | 0,0206 µM |
| NSC-747599 | 50 µM | to | 0,103 µM |
| NSC-747971 | 25 µM | to | 0,103 µM |
| NSC-747972 | 25 µM | to | 0,103 µM |
| NSC-747973 | 25 µM | to | 0,0206 µM |
| NSC-747974 | 25 µM | to | 0,103 µM |
| NSC-749226 | 25 µM | to | 0,103 µM |
| NSC-750 | 50 µM | to | 0,206 µM |
| NSC-750690 | 25 µM | to | 0,103 µM |
| NSC-752 | 50 µM | to | 0,103 µM |
| NSC-754143 | 25 µM | to | 0,0041 µM |
| NSC-754230 | 25 µM | to | 0,000041 µM |
| NSC-755 | 25 µM | to | 0,041 µM |
| NSC-755986 | 50 µM | to | 0,206 µM |
| NSC-756645 | 25 µM | to | 0,041 µM |
| NSC-757441 | 25 µM | to | 0,103 µM |
| NSC-758252 | 25 µM | to | 0,00041 µM |
| NSC-760766 | 25 µM | to | 0,041 µM |
| NSC-761431 | 50 µM | to | 0,103 µM |
| NSC-761432 | 25 µM | to | 0,0021 µM |
| NSC-762 | 50 µM | to | 0,103 µM |
| NSC-77213 | 50 µM | to | 0,206 µM |
| NSC-79037 | 50 µM | to | 0,103 µM |
| NSC-85998 | 50 µM | to | 0,206 µM |
| NSC-8806 | 50 µM | to | 0,103 µM |
| NSC-92859 | 25 µM | to | 0,103 µM |
| NSC-9706 | 25 µM | to | 0,103 µM |

| NSC-102816 | 50 µM | to | 0,103 µM |  | NSC-628503 | 25 µM | to | 0,000041 µM | | NSC-761431 | 50 µM | to | 0,103 µM |
| --- | --- | --- | --- | --- | --- | --- | --- | --- | --- | --- | --- | --- | --- |
| NSC-105014 | 25 µM | to | 0,041 µM |  | NSC-63878 | 25 µM | to | 0,103 µM |  | NSC-761432 | 25 µM | to | 0,0021 µM |
| NSC-109724 | 50 µM | to | 0,206 µM |  | NSC-6396 | 50 µM | to | 0,206 µM |  | NSC-762 | 50 µM | to | 0,103 µM |
| NSC-119875 | 25 µM | to | 0,103 µM |  | NSC-66847 | 25 µM | to | 0,103 µM |  | NSC-77213 | 50 µM | to | 0,206 µM |
| NSC-122758 | 50 µM | to | 0,206 µM |  | NSC-67574 | 25 µM | to | 0,0021 µM | | NSC-79037 | 50 µM | to | 0,103 µM |
| NSC-122819 | 25 µM | to | 0,103 µM |  | NSC-681239 | 25 µM | to | 0,0021 µM | | NSC-85998 | 50 µM | to | 0,206 µM |
| NSC-125066 | 50 µM | to | 0,103 µM |  | NSC-686673 | 50 µM | to | 0,206 µM |  | NSC-8806 | 50 µM | to | 0,103 µM |
| NSC-125973 | 25 µM | to | 0,0021 µM |  | NSC-698037 | 50 µM | to | 0,103 µM |  | NSC-92859 | 25 µM | to | 0,103 µM |
| NSC-127716 | 50 µM | to | 0,206 µM |  | NSC-701852 | 25 µM | to | 0,103 µM |  | NSC-9706 | 25 µM | to | 0,103 µM |
| NSC-13875 | 50 µM | to | 0,206 µM |  | NSC-702294 | 50 µM | to | 0,206 µM |  |  |  |  |  |
| NSC-138783 | 50 µM | to | 0,206 µM |  | NSC-712807 | 50 µM | to | 0,206 µM |  |  |  |  |  |
| NSC-1390 | 50 µM | to | 0,206 µM |  | NSC-713563 | 50 µM | to | 0,103 µM |  |  |  |  |  |
| NSC-141540 | 50 µM | to | 0,103 µM |  | NSC-71423 | 50 µM | to | 0,206 µM |  |  |  |  |  |
| NSC-14229 | 25 µM | to | 0,041 µM |  | NSC-715055 | 25 µM | to | 0,0021 µM | |  |  |  |  |
| NSC-169780 | 50 µM | to | 0,206 µM |  | NSC-718781 | 25 µM | to | 0,041 µM |  |  |  |  |  |
| NSC-180973 | 25 µM | to | 0,103 µM |  | NSC-719276 | 50 µM | to | 0,103 µM |  |  |  |  |  |
| NSC-18509 | 25 µM | to | 0,103 µM |  | NSC-719344 | 50 µM | to | 0,206 µM |  |  |  |  |  |
| NSC-19893 | 50 µM | to | 0,103 µM |  | NSC-719345 | 50 µM | to | 0,206 µM |  |  |  |  |  |
| NSC-218321 | 50 µM | to | 0,206 µM |  | NSC-719627 | 50 µM | to | 0,103 µM |  |  |  |  |  |
| NSC-226080 | 50 µM | to | 0,103 µM |  | NSC-721517 | 50 µM | to | 0,206 µM |  |  |  |  |  |
| NSC-241240 | 50 µM | to | 0,206 µM |  | NSC-732517 | 25 µM | to | 0,0041 µM | |  |  |  |  |
| NSC-25154 | 50 µM | to | 0,206 µM |  | NSC-733504 | 25 µM | to | 0,103 µM |  |  |  |  |  |
| NSC-26271 | 50 µM | to | 0,206 µM |  | NSC-737754 | 25 µM | to | 0,103 µM |  |  |  |  |  |
| NSC-266046 | 50 µM | to | 0,103 µM |  | NSC-740 | 25 µM | to | 0,0021 µM | |  |  |  |  |
| NSC-26980 | 25 µM | to | 0,0206 µM |  | NSC-743414 | 50 µM | to | 0,206 µM |  |  |  |  |  |
| NSC-27640 | 25 µM | to | 0,0021 µM |  | NSC-745750 | 25 µM | to | 0,0206 µM | |  |  |  |  |
| NSC-296961 | 50 µM | to | 0,206 µM |  | NSC-747599 | 50 µM | to | 0,103 µM |  |  |  |  |  |
| NSC-3088 | 25 µM | to | 0,103 µM |  | NSC-747971 | 25 µM | to | 0,103 µM |  |  |  |  |  |
| NSC-312887 | 50 µM | to | 0,206 µM |  | NSC-747972 | 25 µM | to | 0,103 µM |  |  |  |  |  |
| NSC-32065 | 50 µM | to | 0,206 µM |  | NSC-747973 | 25 µM | to | 0,0206 µM | |  |  |  |  |
| NSC-34462 | 50 µM | to | 0,103 µM |  | NSC-747974 | 25 µM | to | 0,103 µM |  |  |  |  |  |
| NSC-362856 | 50 µM | to | 0,206 µM |  | NSC-749226 | 25 µM | to | 0,103 µM |  |  |  |  |  |
| NSC-369100 | 50 µM | to | 0,206 µM |  | NSC-750 | 50 µM | to | 0,206 µM |  |  |  |  |  |
| NSC-38721 | 50 µM | to | 0,103 µM |  | NSC-750690 | 25 µM | to | 0,103 µM |  |  |  |  |  |
| NSC-409962 | 50 µM | to | 0,206 µM |  | NSC-752 | 50 µM | to | 0,103 µM |  |  |  |  |  |
| NSC-45388 | 50 µM | to | 0,206 µM |  | NSC-754143 | 25 µM | to | 0,0041 µM | |  |  |  |  |
| NSC-45923 | 50 µM | to | 0,206 µM |  | NSC-754230 | 25 µM | to | 0,000041 µM | |  |  |  |  |
| NSC-49842 | 25 µM | to | 0,0021 µM |  | NSC-755 | 25 µM | to | 0,041 µM |  |  |  |  |  |
| NSC-606869 | 50 µM | to | 0,103 µM |  | NSC-755986 | 50 µM | to | 0,206 µM |  |  |  |  |  |
| NSC-608210 | 25 µM | to | 0,00021 µM |  | NSC-756645 | 25 µM | to | 0,041 µM |  |  |  |  |  |
| NSC-609699 | 25 µM | to | 0,206 µM |  | NSC-757441 | 25 µM | to | 0,103 µM |  |  |  |  |  |
| NSC-613327 | 25 µM | to | 0,0021 µM |  | NSC-758252 | 25 µM | to | 0,00041 µM | |  |  |  |  |
| NSC-616348 | 50 µM | to | 0,103 µM |  | NSC-760766 | 25 µM | to | 0,041 µM |  |  |  |  |  |

**Figure S1**


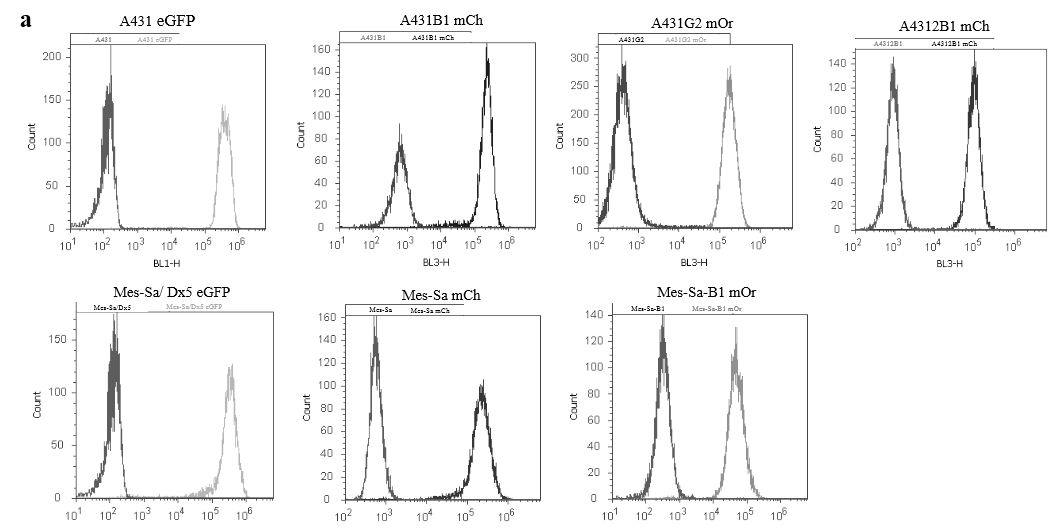


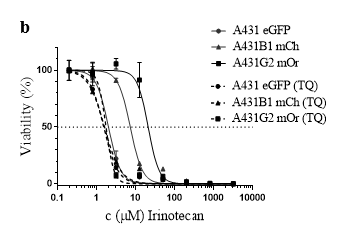

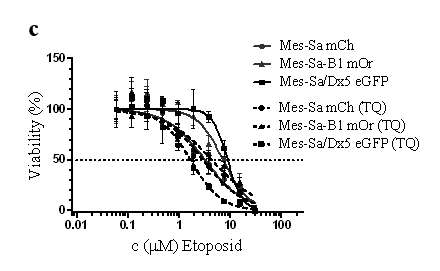


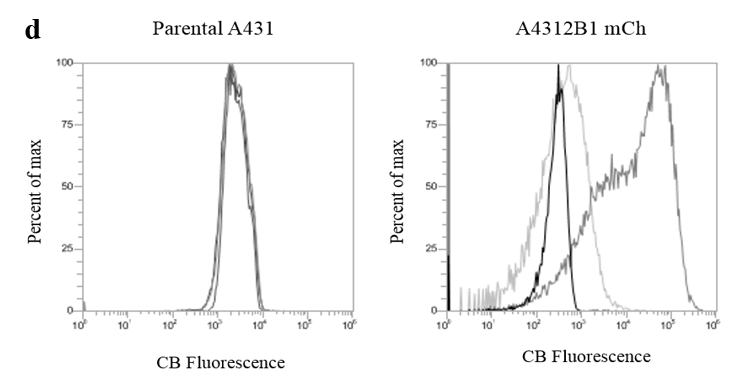


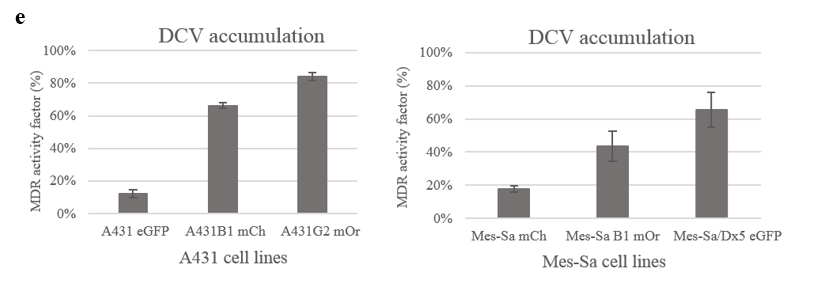


**Figure S2**

**Figure S3**


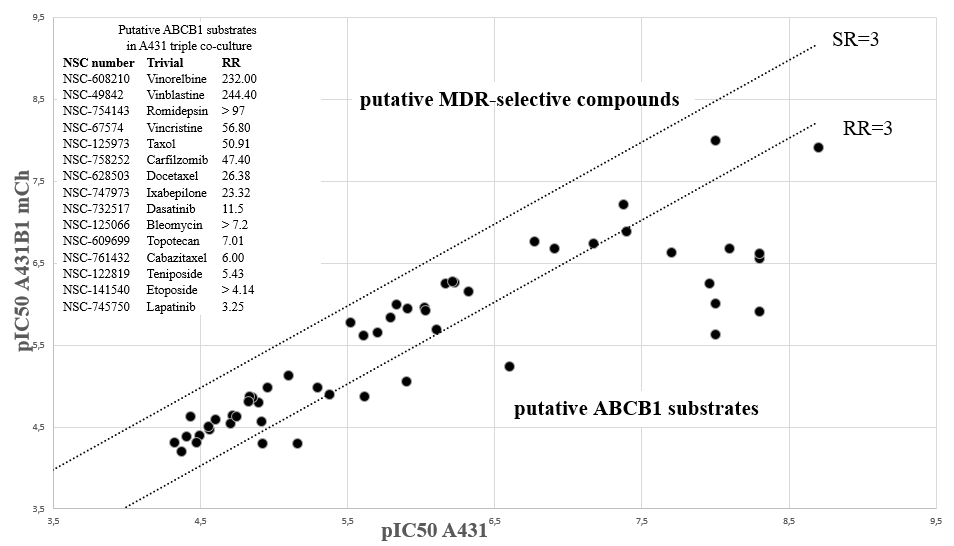


**Figure S4:**


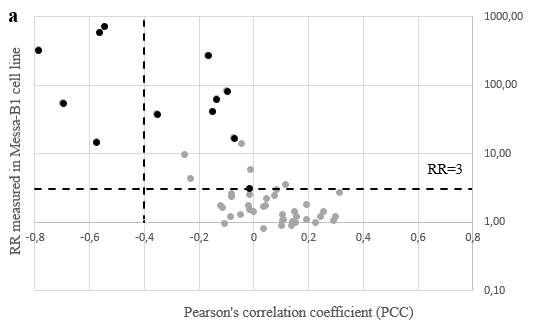


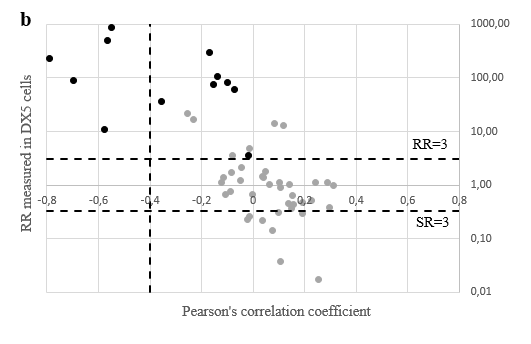


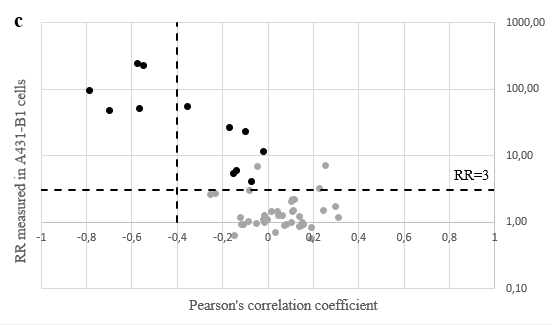

Supplement: Supplementary file 1 — Supplementary material 1 (DOCX 407 KB) [file 204_2019_2417_MOESM1_ESM.docx]
